# Immunoendocrine Insights Into Dehydroepiandrosterone by Cytokine Profiling in Sub-fertile Women Undergoing Intrauterine Insemination

**DOI:** 10.7759/cureus.86899

**Published:** 2025-06-28

**Authors:** Shamaila Mahboob, Kashaf Qayyum, Shama Chaudhry, Zareena Begum

**Affiliations:** 1 Gynecology, Services Hospital, Lahore, PAK; 2 Obstetrics and Gynecology, Pakistan Institute of Medical Sciences, Islamabad, PAK; 3 Obstetrics and Gynecology, Dr. Ziauddin University Karachi, Karachi, PAK; 4 Obstetrics and Gynecology, Saidu Group of Teaching Hospitals, Saidu Sharif Swat, Swat, PAK

**Keywords:** cytokines, dhea, elisa, il-10, il-6, intrauterine insemination, subfertility, tnf-α

## Abstract

Background: Immunological and hormonal problems, notably changes in cytokine levels, are now being closely linked to sub-fertility. This study aimed to assess how sub-fertile women respond to intrauterine insemination (IUI) after dehydroepiandrosterone (DHEA) treatment and compare them to healthy fertile women in terms of cytokine profiles.

Methods: A total of 90 participants took part in the cross-sectional study: 60 sub-fertile women taking DHEA (25 mg thrice daily for eight weeks) and 30 healthy fertile controls. Enzyme-linked immunosorbent assay (ELISA) was used to determine the amounts of interleukin-6 (IL-6), tumor necrosis factor-alpha (TNF-α), and interleukin-10 (IL-10) present in the serum samples. Data was analyzed with the help of Statistical Package for the Social Sciences (SPSS) version 25.0 (IBM Corp., Armonk, NY). A comparison of cytokine levels was made using an independent t-test, with p < 0.05 considered significant.

Results: Mean IL-6 values in sub-fertile women (12.8 ± 4.1 pg/mL) were substantially higher than in the control group (7.2 ± 2.5 pg/mL) (p = 0.001). Patients in the study had TNF-α levels of 18.5 ± 5.3 pg/mL, higher than the control group with 11.7 ± 3.8 pg/mL (p = 0.003). Our results showed that interleukin-10 (IL-10) was significantly lower in the sub-fertile group (6.4 ± 2.2 pg/mL) than in the control group (10.1 ± 2.9 pg/mL) (p = 0.002). The findings revealed that sub-fertile women continue to show a pro-inflammatory response in their immune system.

Conclusion: Sub-fertile women on DHEA and treated with IUI had higher levels of pro-inflammatory cytokines and lower levels of anti-inflammatory cytokines than did healthy controls. The results suggested that certain immunological responses could help, and more research was needed to learn how cytokines influence fertility management.

## Introduction

Approximately 15% of couples worldwide struggle with sub-fertility, which is now seen as a result of a combination of hormone imbalances, immune system dysfunction, and reproduction issues [1]. Studies have demonstrated that issues with cytokines negatively affect both the ability of the endometrium to receive the embryo and the implantation of that embryo [2]. Being in an inflammatory state with interleukin-6 (IL-6) and tumor necrosis factor-alpha (TNF-α) at higher levels and cytokines such as interleukin-10 (IL-10) at lower levels can cause reproductive problems, either preventing conception or causing an early loss of the pregnancy [3].

Given this immunoendocrine imbalance, investigations are underway to discover drugs that can restore cytokine stability [4]. Dehydroepiandrosterone (DHEA), a hormone produced in the adrenal glands, is now being used successfully in combination with fertility treatments [5]. Although initially, it was suggested to boost fertility by preserving ovarian cells, recent studies suggested that DHEA plays a role in the immune system as well and may change the way cytokines are expressed [6]. In prior clinical trials, it is indicated that DHEA can boost the regulation by T-cells and affect the ratio of pro-inflammatory to anti-inflammatory cytokines [7].

However, the effect of DHEA on the immune system during human fertility, particularly in sub-fertile women undergoing intrauterine insemination (IUI), is not well explored yet. The study was designed to measure the levels of IL-6, TNF-α, and IL-10 in the serum of both sub-fertile women taking DHEA and fertile controls. This study supposed that if DHEA affects immune system balance, it would bring cytokine expression in sub-fertile women toward normal ranges.

## Materials and methods

This study used a cross-sectional approach to evaluate the effects of dehydroepiandrosterone (DHEA) supplementation on serum cytokine profiles in sub-fertile women undergoing intrauterine insemination (IUI), in comparison to healthy fertile women. The study was conducted and analyzed in the Pakistan Institute of Medical Sciences, Ziauddin University, and Services Hospital Lahore (14/03/25/1425MS). Among the total of 90 women, 60 were sub-fertile and given DHEA, and 30 were healthy and fertile women. The study included participants who were seen in the outpatient department of a tertiary care fertility clinic over a period of six months. All participants gave informed consent before enrollment. The groups were assigned using a random sampling technique. Sample size was computed using the OpenEpi version 3.0.0 (released 2013, Atlanta, GA, USA), with a 95% confidence interval and a 5% chance of error [8].

Participants were sub-fertile women aged 20-40 years who had unexplained sub-fertility or fewer egg production in the ovaries and were planned for treatment with IUI. Only those women with normal menstrual cycles and no known autoimmune or endocrine problems were enrolled in the study. A woman in the fertile control group was chosen only if she had at least one natural conception in the past two years and had not experienced infertility. Participants were excluded if they had used hormonal or immunomodulatory drugs in the last three months, had current or recent infections, suffered from systemic inflammatory or autoimmune diseases, or had chronic diseases such as diabetes, high blood pressure, or thyroid dysfunction.

Before the IUI cycle, sub-fertile participants received DHEA orally at a dose of 25 mg, taken three times daily for eight weeks to make a total daily dose of 75 mg. Adherence to the treatment was checked using patients’ records of their supplements and by having them come in for regular follow-up visits. After the eighth week, DHEA treatment was completed, and blood samples were taken from both groups in the early follicular phase (days 2-4) of their menstrual cycle. The serum was separated from the blood using centrifugation and placed in a -80°C freezer until it was studied. Each serum sample was tested for cytokine levels IL-6, TNF-α, and IL-10 by using commercially available enzyme-linked immunosorbent assay (ELISA) kits according to the manufacturer’s protocol. The data were analyzed using Statistical Package for the Social Sciences (SPSS) version 25.0 (IBM Corp., Armonk, NY). Cytokine concentrations were measured as the mean value ± the standard deviation (SD) using an independent t-test, with p < 0.05 considered significant.

## Results

The study included 90 women, of whom 60 were sub-fertile and the other 30 were fertile. Demographic variables such as body mass index (BMI) and education level were comparable between groups. Clinically, sub-fertile women had significantly lower anti-Müllerian hormone (AMH) and higher follicle-stimulating hormone (FSH) levels. Table 1 shows the basic information about the study participants.

**Table 1:** Demographic Characteristics of the Study Participants Descriptive statistics used; p < 0.05 considered significant BMI: body mass index, SD: standard deviation, N/A: not applicable, n: sample size, %: percentage, kg/m²: kilogram per meter square

Variable	Sub-fertile group (n = 60)	Control group (n = 30)	p-value
Age (years, mean ± SD)	31.6 ± 4.5	30.9 ± 4.2	0.48
BMI (kg/m², mean ± SD)	25.3 ± 3.1	24.8 ± 2.9	0.37
Duration of infertility (years)	4.2 ± 2.1	N/A	N/A
Parity (nulliparous, %)	60 (100%)	0%	N/A
Education ≥ 12 years (%)	42 (70%)	46 (76.7%)	0.49

The average age of participants was 31.6 ± 4.5 years among those with sub-fertility and 30.9 ± 4.2 years among the normal group, and there was no significant difference (p = 0.48). Both groups had similar BMI results and the same educational background of ≥12 years (42 (70%) versus 46 (76.7%); p = 0.49), suggesting no significant difference. The hormone levels are compared between the study groups in Table 2.

**Table 2:** Clinical and Hormonal Profile of the Participants Independent t-test used; p < 0.05 considered significant AMH: anti-Müllerian hormone, FSH: follicle-stimulating hormone, LH: luteinizing hormone, DHEA: dehydroepiandrosterone, SD: standard deviation, N/A: not applicable, pg/mL: picograms per milliliter, ng/mL: nanograms per milliliter, mIU/mL: milli-international units per milliliter

Variable	Sub-fertile (n = 60)	Control (n = 30)	Test used	Test value	p-value
AMH (ng/mL, mean ± SD)	1.4 ± 0.7	2.6 ± 1.1	t-test	t = -5.82	0.001
FSH (mIU/mL, mean ± SD)	9.1 ± 3.2	6.3 ± 2.4	t-test	t = 4.15	0.003
LH/FSH ratio	1.3 ± 0.4	0.9 ± 0.2	t-test	t = 3.12	0.01
Estradiol (pg/mL)	62.5 ± 18.4	77.3 ± 15.1	t-test	t = -3.26	0.002
DHEA usage duration (weeks)	8.0 ± 0.0	N/A	N/A	N/A	N/A

Hormonal levels were significantly different in the sub-fertile group than in the control group. There was a significant drop in anti-Müllerian hormone (AMH) (p = 0.001), along with a significant increase in follicle-stimulating hormone (FSH) (p = 0.003), which suggests that the ovarian reserve is decreased. The cytokine levels of the two groups are displayed in Table 3.

**Table 3:** Cytokine Profiles of the Study Participants Independent t-test used; p < 0.05 considered significant IL-6: interleukin-6, TNF-α: tumor necrosis factor-alpha, IL-10: interleukin-10, SD: standard deviation, pg/mL: picograms per milliliter, n: sample number

Cytokine	Sub-fertile (n = 60)	Control (n = 30)	Test used	Test value	p-value
IL-6 (pg/mL)	12.8 ± 4.1	7.2 ± 2.5	t-test	t = 7.12	0.001
TNF-α (pg/mL)	18.5 ± 5.3	11.7 ± 3.8	t-test	t = 6.26	0.003
IL-10 (pg/mL)	6.4 ± 2.2	10.1 ± 2.9	t-test	t = -5.93	0.002

## Discussion

The study aimed to understand the effects of DHEA on the immune and endocrine systems in sub-fertile women receiving intrauterine insemination (IUI), by measuring cytokines and hormones. Our results showed that even with DHEA supplementation, sub-fertile women continued to have too many pro-inflammatory cytokines (IL-6 and TNF-α) and too few anti-inflammatory ones (IL-10), which may make it difficult for them to implant. This suggested that difficulties with the immune response could contribute to sub-fertility and might not be solved only by hormone treatments [9,10]. A study found that taking DHEA improved ovarian reserve factors such as AMH and FSH in women with diminished ovarian reserve (DOR), but its effect on general inflammation was not consistent [11]. Although DHEA improved AMH and FSH in our study, inflammation in the cytokines remained unchanged, suggesting that DHEA does not control inflammation effectively.

In another study, DHEA supplementation resulted in better cycle outcomes for poor responders, but the study did not examine any changes in immunological markers [12]. However, our study demonstrated that although endocrine health was better, high levels of IL-6 and TNF-α in the immune system still existed. According to one study, there was more oxidative stress and higher inflammatory cytokines in the follicular fluid of women with DOR, just as we noticed elevated IL-6 and TNF-α in serum [13]. Our findings confirmed the earlier suggestion that chronic inflammation was a common factor in sub-fertility. Additionally, one study discovered that providing DHEA increased ovarian function and changed certain immune responses there, but its effect on the systemic immune system was not significant [14].

We found the same as they did, showing that even with improved ovarian hormones, systemic cytokine levels might not go back to normal. Higher IL-6 levels in infertile women receiving assisted reproductive technology (ART) were connected to a lower chance of implantation and pregnancy in some studies [15]. This matched our findings, since higher IL-6 in sub-fertile women could reduce their endometrium’s ability to accept the embryo. One study showed that women facing unexplained infertility had higher TNF-α levels and lower IL-10 levels than fertile women [16,17].

However, there were some limitations to this study despite its valuable results. Because of its cross-sectional design, it was not possible to draw direct links between DHEA use and changes in immune factors. Since the cytokine panel was only made up of three markers, future studies should contain more immunological factors. Analyzing the immune system in follicular fluid or endometrial tissue might help us find out how it works. Furthermore, using DHEA along with anti-inflammatory substances could represent a new strategy to improve fertility in women with immune problems.

## Conclusions

This study showed that taking DHEA might help improve important hormones such as AMH and FSH in sub-fertile women, which could suggest a partial improvement in ovarian function. Although hormones were better controlled, the cytokine levels were still pro-inflammatory, pointing to ongoing immune imbalance.

Since there was increased IL-6 and TNF-α and reduced IL-10, it seemed that DHEA alone might not handle all the immunological problems that cause sub-fertility. The research suggested that using immune-targeted therapies together with hormonal help could be useful in managing sub-fertility in women with underlying inflammatory problems.
